# Protein-coated pH-responsive gold nanoparticles: Microwave-assisted synthesis and surface charge-dependent anticancer activity

**DOI:** 10.3762/bjnano.5.158

**Published:** 2014-09-04

**Authors:** Dickson Joseph, Nisha Tyagi, Christian Geckeler, Kurt E.Geckeler

**Affiliations:** 1 Department of Nanobio Materials and Electronics (WCU), Gwangju Institute of Science & Technology (GIST), Gwangju 500-712, South Korea; 2 Department of Materials Science and Engineering, Gwangju Institute of Science & Technology (GIST), Gwangju 500-712, South Korea; 3 Institute of Medical System Engineering, Gwangju Institute of Science & Technology (GIST), Gwangju 500-712, South Korea

**Keywords:** anticancer, cytotoxicity, gold Nanoparticles, pH, protein, zeta potential

## Abstract

The biocompatibility and ease of functionalization of gold nanoparticles underlie significant potential in biotechnology and biomedicine. Eight different proteins were examined in the preparation of gold nanoparticles (AuNPs) in aqueous medium under microwave irradiation. Six of the proteins resulted in the formation of AuNPs. The intrinsic pH of the proteins played an important role in AuNPs with strong surface plasmon bands. The hydrodynamic size of the nanoparticles was larger than the values observed by TEM and ImageJ. The formation of a protein layer on the AuNPs accounts for this difference. The AuNPs exhibited sensitivity towards varying pH conditions, which was confirmed by determining the difference in the isoelectric points studied by using pH-dependent zeta potential titration. Cytotoxicity studies revealed anticancerous effects of the AuNPs at a certain micromolar concentration by constraining the growth of cancer cells with different efficacies due to the use of different proteins as capping agents. The positively charged AuNPs are internalized by the cells to a greater level than the negatively charged AuNPs. These AuNPs synthesized with protein coating holds promise as anticancer agents and would help in providing a new paradigm in area of nanoparticles.

## Introduction

Blending nanotechnology with biomaterials has received keen attention due to a growing need to develop environmentally benign technologies by applying green chemistry principles towards greener nanomaterial syntheses. For metal or metal oxide nanoparticles targeted towards biomedical applications, emphasis is put on the development of protocols which involve green chemistry and do not comprise toxic chemicals in the synthesis procedures to avoid adverse effects during applications [1–3]. Metallic nanoparticles show promise in applications such as catalysis, electronics, chemical labeling, optics and sensors. [4–8]. Nanomaterials that are biocompatible have gained research interest for biomedical applications that include cancer treatment [9–10]. Consequently, one of the challenges in synthesizing nanoscale biocompatible materials is designing methodologies with biological molecules as the templates. Towards this purpose, biomolecules such as proteins/enzymes and DNA possess significant advantage as both reducing and capping agents on gold, which is the most extensively studied system in this category [11–12]. Gold nanoparticles are known for their unique characteristics that include precise size control and ease of fuctionalisation [13–15]. The change in color of colloidal gold solutions provides information about processes that take place in the nano-size range, which has been used in detection studies based on the study by Mirkin et. al [16].

Biomolecules have been reported to interact with gold salts and reduce them into metallic gold, acting both as a reductant and stabilizer [12,17–21]. Proteins, such as bovine serum albumin, silk fibroin protein, chicken egg white lysozyme, α-amylase, green fluorescent protein and apoferritin have been investigated in the synthesis of AuNPs [12,18]. However, the reducing and capping capabilities of different proteins using a single protocol for the synthesis of any nanoparticles has not been explored or reported. In our previous study, four kinds of surfactants were utilized to study their influence on the shape of the nanoparticle [22]. We observed that, by using a single protocol, we could obtain four different gold nanostructures depending on the surfactant used for the capping. For this purpose sodium dodecyl sulfate (SDS), cetyl trimethylammonium bromide (CTA), *N*-dodecyl-*N*,*N*-dimethyl-3-ammonio-1-propanesulfonate (DAP), and Tween-80 (T80) were utilized. Leaf-like, rugged, dendritic, and tadpole-shaped gold nanostructures were formed in the presence of SDS, CTA, DAP and T80, respectively. Here, we have attempted to explore the ability of eight different lyophilized proteins with differences in molecular weights, isoelectric points (IEP), and functions for the preparation of AuNPs. The AuNPs were prepared by using a one-pot green synthetic protocol assisted by microwave irradiation in the presence of proteins in aqueous media. Because proteins with different IEPs have been utilized for the capping of AuNPs, the resulting AuNPs were expected to behave differently under variable pH conditions [23]. Hence, pH-dependent zeta potential titration studies were performed to determine the surface charge of the AuNPs at different pH conditions.

Cancer is one of the leading causes of early deaths in the modern world. Although significant advances in science and technology have given us a more comprehensive understanding of cancer, the diagnosis and treatment of cancer remain challenging. The inability of many anticancer drugs to reach the specific target sites and their common systemic delivery is a major concern, because their unspecificity hampers the growth of normal tissues and cells. The advent of nanotechnology offers a wide range of tools for the diagnosis and the treatment of this deadly disease, with new imaging techniques and the targeted delivery of therapeutic agents offering lower costs and minimal side effects [24]. Because of their biocompatibility, noble metal nanoparticles, particularly AuNPs, are more preferable in various biomedical applications, including highly sensitive diagnostic assays [25], thermal ablation, radiotherapy enhancement [26–28], as well as for targeted drug and gene delivery [29–31]. Hence, MTT assays were used to study the cell viabilities of fibroblasts and cancer cells after treatment with AuNPs to check the cytotoxicity and the anticancer properties of the AuNPs for their future biomedical applications.

## Results and Discussion

### Synthesis and characterization of gold nanoparticles prepared by using proteins

The objective of this work was to rapidly prepare AuNPs in an aqueous medium by using the greenest protocol possible to examine the effect of proteins, which are natural polyampholytes that possess different isoelectric points, on the properties of the AuNPs. In this regard, eight different proteins with diverse molecular weights, isoelectric points, and functions were chosen (Table 1) and used in the studies. The optimum experimental conditions for the preparation of AuNPs were standardized by choosing bovine hemoglobin (BHG) with a molecular weight of 62,108 g/mol [32] as the standard protein, and the optimized procedure was adopted for the other proteins.

**Table 1:** Molar mass, isoelectric point and function of different proteins.

protein	molar mass (g/mol)	IEP	no of disulfide bridges	function

HIS	13,942	11.8	0	A chief protein component of chromatin, which acts as spools around which DNA winds, and they play a role in gene regulation.
LYS	14,331	10.7	4	An enzyme that helps in breaking down the polysaccharide walls of many kinds of bacteria
TRY	23,783	9.2	6	A serine protease, which hydrolyses proteins.
OVA	42,952	4.6	1	A storage protein in chicken egg white
BHG	62,108	9.4	0	A metelloprotein that helps in the oxygen transport.
BSA	66,562	4.7	17	A plasma protein that helps ot maintain the osmotic pressure.
GOX	131,276	4.2	1	An enzyme that helps in the breakdown of sugars.
BGG	156,000	6.7	n/a	A plasma protein that acts as antibody defense against antigen invasion.

The standardization experiments were based on BHG because of two main reasons. First, its molecular weight is between the lowest and highest molecular weights of the eight selected proteins in Table 1. Second, because HG exists as a single complex with a molecular weight greater than 60,000 g/mol in its natural state and its four polypeptide chains are separated (each with molecular weights of approximately 17,000 g/mol) in its denatured state, thus, the natural and denatured states have distinct characteristics. Based on these two factors, optimizing the experimental parameters by using BHG would be the best approach for devising a single procedure applicable to the other proteins.

The reactions were carried out at 25 °C. The protein and Au salt concentration were 1 mg/mL and 0.5 mM, respectively. These conditions did not result in the formation of AuNPs. The addition of Ag ions to a mixture containing Au ions has been reported to form AuNPs in the presence of bovine serum albumin (BSA) [33]. Hence, we added AgNO_3_ at a ratio of 5:1 between the Au and Ag ions and observed a red color that is characteristic for the formation of AuNPs. Several experiments were carried out by varying the thermal conditions but the AuNP formation took several hours. Hence, we used microwave irradiation with an aim to reduce the reaction time, as it is known to accelerate chemical reactions. To standardize the microwave reaction conditions, several experiments were conducted in which the irradiation power, temperature and time were varied, and the products were analyzed by UV–vis spectroscopy. A microwave power of 250 W and a temperature of 120 °C were found to be optimum conditions to obtain highly stable AuNPs with a best yield in a time frame of 10 min. When the power was greater than 250 W, there was no significant change in the UV absorbance. However, if the temperature exceeded 120 °C, then the UV absorbance intensity decreased, and the AuNPs eventually became unstable. In terms of the reaction time, the maximum UV absorbance was achieved after 6 min, but we carried out the reaction for approximately 10 min to reduce any unreduced Au ions that may have remained in the solution.

**Figure 1:**
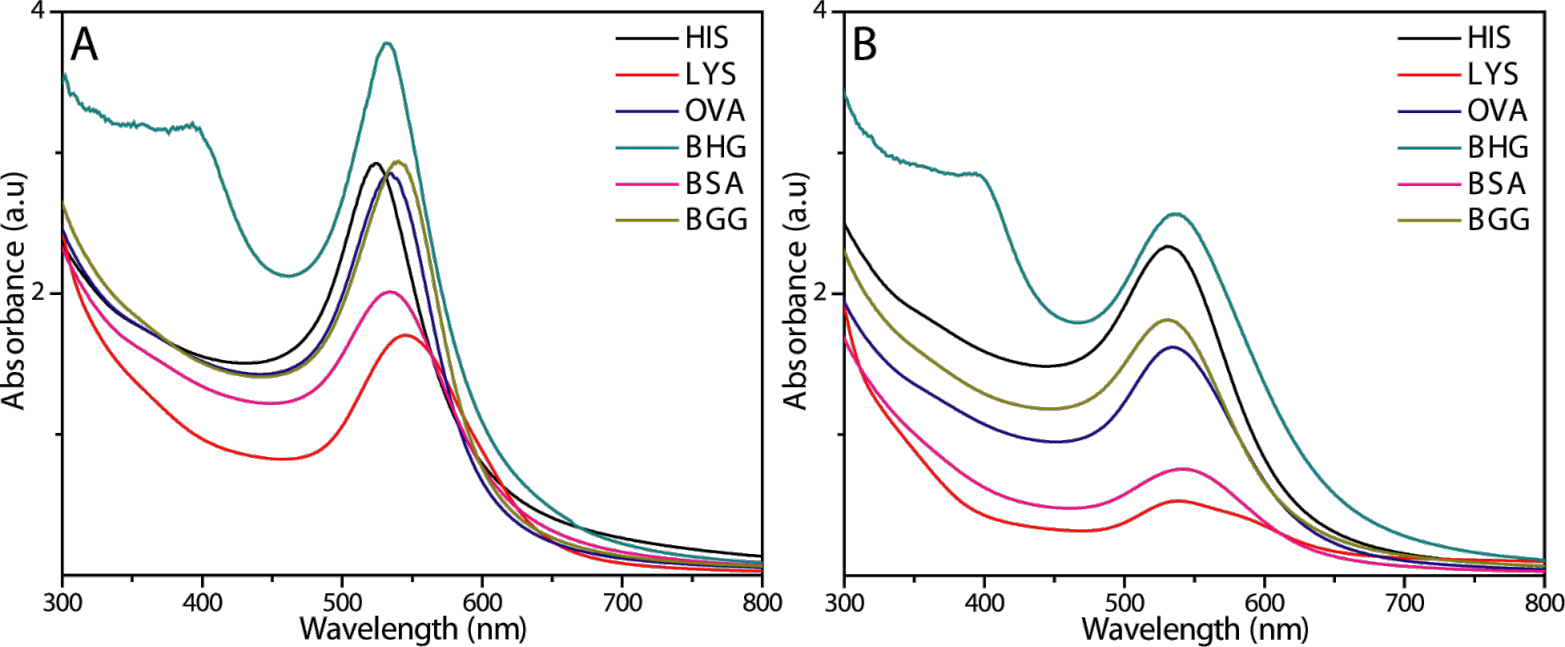
UV–vis spectra of the AuNPs (0.5 mM) prepared using different proteins (A) with and (B) without Ag ions at their intrinsic pH.

**Figure 2:**
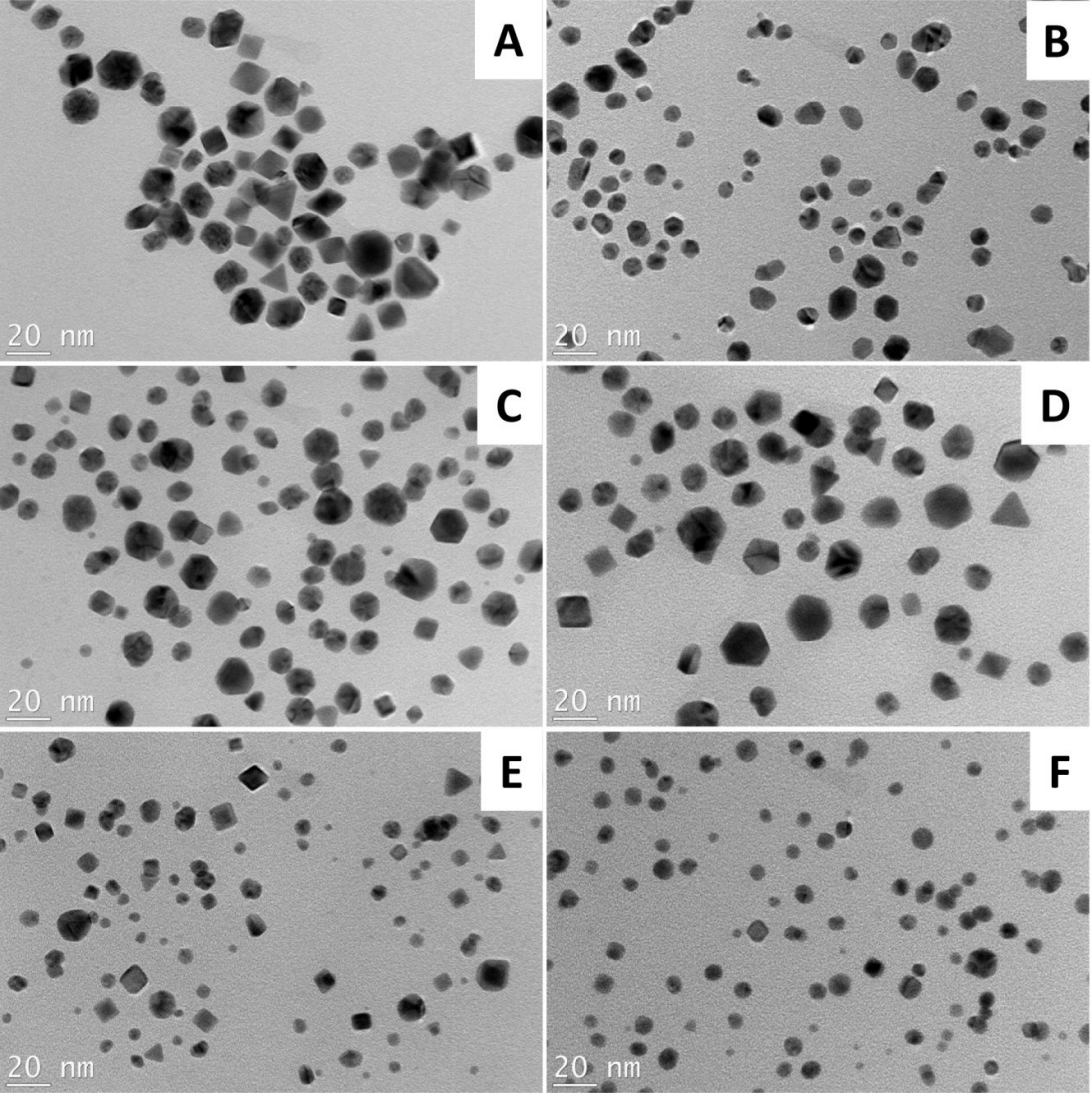
TEM images of the AuNPs (0.5 mM) prepared by using different proteins: (A) HIS, (B) LYS, (C) OVA, (D) BHG, (E) BSA and (F) BGG. Scale bar: 20 nm.

The standardized procedure was employed for the other proteins listed in Table 1, and the reactions were carried out with and without Ag ions. The protein concentrations were 1 mg/mL with Au and Ag salt concentrations of 0.5 and 0.1 mM, respectively. The samples were irradiated with a microwave power of 250 W at 120 °C for 10 min. A wine red color that is characteristic of AuNP formation was observed after the reaction for all of the proteins except trypsin (TRY) and glucose oxidase (GOX). The AuNPs formed in the presence of the different proteins were characterized by UV–vis spectroscopy (Figure 1). The addition of Ag ions played an important role in the formation of the AuNPs, which is evident in the UV–vis spectra. The AuNPs prepared by the addition of Ag ions showed intense and narrow UV absorbance peaks in comparison to the AuNPs without Ag ions, which suggests that the Ag ions play a vital part during the conversion of Au ions into AuNPs. The reactions were also carried out with the different proteins by adding only Ag ions to study the formation of AgNPs during microwave irradiation. Surprisingly, no AgNPs were formed with any of the protein samples based on the absence of UV absorbance at approximately 400 nm, which is characteristic of AgNPs. Although no AgNPs were observed at an Ag ion concentration of 0.1 mM, AgNPs were formed at higher concentrations. A yellow coloration and an UV absorbance near 400 nm suggested that AgNPs were formed for all of the proteins samples except for TRY and GOX. Hence, the presence of Ag ions in the reaction mixture might result in a very low concentration of AgNPs, which are consumed in a galvanic exchange reaction converting Au ions to AuNPs at a faster rate [33–34]. From the UV–vis spectra (Figure 1B) for the AuNPs formed in the absence of Ag ions, the peaks were broader because the reaction is slow, which results in larger AuNPs. However, when the optimum amount of AgNO_3_ was added, the peaks were narrow, indicating that all of the formed AgNPs were consumed at the same time to form AuNPs with a uniform and narrow size distribution. Equal or higher concentrations of Ag ions relative to Au ions resulted in irregularly shaped AuNPs, which became unstable. The prepared AuNPs with different proteins were characterized by TEM, DLS and ImageJ to study their morphology and size. The hydrodynamic size of the AuNPs was larger than the size measured from the TEM images. This difference in size results from the presence of the protein layers covering the AuNPs that were not observed in the TEM images but in the DLS measurements (Table S2, Supporting Information File 1) [35]. Figure 2 shows the TEM images of the corresponding protein-coated AuNPs.

In addition, the six different proteins that formed AuNPs at the intrinsic pH were selected, and the reactions were carried out by modifying the pH of the initial reactant mixture. These studies were performed at acidic, neutral and basic pH conditions to determine the influence of pH on the synthesis of the AuNPs. The intrinsic pH of the protein–metal salts reactant solution was altered by the addition of 1 N HCl or 1 N NaOH. The UV–vis spectroscopic studies revealed that the intrinsic pH of the protein led to the formation of AuNPs with strong surface plasmon resonance (SPR) bands. However, at acidic, neutral and basic pH conditions, either AuNPs with weak SPR bands or no AuNPs were formed (Figure S1, Supporting Information File 1). Photographs of the dispersions (Figure S2, Supporting Information File 1) were taken after the microwave irradiation under different pH conditions, and the color characteristic of the AuNPs was observed only for reactions carried out at the intrinsic pH values. Whereas at acidic, neutral and basic pH conditions, different colored dispersions were observed, and detailed studies on their size and shape are in progress. Hence, varying the pH of the protein solution modifies the structural confirmation of the protein, which alters the native state of the amino acids and thereby deactivates some of the amino acid residues responsible for the reduction of the Au ions.

### Structural conformation

The main objective of this work was the preparation of AuNPs by using proteins, not retaining the structural conformation of the proteins, because changes in the structural conformations of the proteins were expected upon microwave irradiation. Yet, we were curious to study the structural conformation of the protein, so the following characterization studies were conducted. First, UV–vis spectroscopic studies were carried out on blank protein samples that were microwave irradiated and non-irradiated. Based on Figure S3 (Supporting Information File 1), we observed that non-globular monomer proteins, such as lysozyme (LYS) and histone (HIS), did not undergo any change in their UV absorbance, whereas globular monomer proteins, such as ovalbumin (OVA) and BSA, showed an increase in the UV intensity and an absence of the sharp peak at 279 and 278 nm, respectively. BHG, a globular tetramer containing four polypeptides, and bovine gamma globulin (BGG), a dimer with two polypeptides, showed decreases in their UV absorbance and complete disappearance of the peaks at 405 and 278 nm, respectively. Based on these results, we speculate that there were no major structural deformations for LYS and HIS. However, OVA and BSA, which are globular proteins, may have unpacked into a linear chain, thereby exposing other amino acids that could absorb in the UV region and thus increasing the UV intensity that could lead to a change in the structural conformation. The polypeptides in BHG and BGG that are non-covalently bound are separated under microwave irradiation into individual linear chain monomer polypeptides, which causes a large structural deformation that is observed by the drastic change in the UV absorbance of the respective proteins. From these studies, the structural conformation remains intact for LYS and HIS, but the other four proteins undergo a major change upon microwave irradiation. Similar UV studies were not possible with the AuNPs samples because the AuNP absorbance masks the absorbance of the proteins.

Second, FTIR vibrational frequencies of the proteins and AuNPs were investigated to compare the secondary structural conformation of the proteins in the AuNPs and the blank proteins. The amide group vibrations of the protein provide information on their secondary structure [36]. The FTIR spectra of the pure BSA and the BSA–AuNPs pellets were measured because the IR spectrum of BSA has been well studied [37–38]. The bands corresponding to amide-I, amide-II and amide-III at 1663 cm^−1^, 1541 cm^−1^ and 1247 cm^−1^, respectively, for BSA–AuNP are more prominent than BSA with shifts in the peaks, confirming that the secondary structure of the protein is intact (Figure S4, Supporting Information File 1). Similar results were also observed for the other protein–AuNPs. The FTIR spectra for the LYS and BGG samples are shown in Figure S4 (Supporting Information File 1).

Lastly, SDS-PAGE studies were conducted to determine the concentration of the proteins and to understand their primary structure. The results for BSA and the BSA–AuNPs are shown in Figure S5 (Supporting Information File 1). A strong band at 66,562 g/mol is observed for BSA, whereas the BSA–AuNPs showed a weak band at the same molecular weight. The weak band for the BSA–AuNPs reveals that some of the proteins are hydrolyzed during the microwave irradiation step [39–40], leading to a decrease in the overall concentration of BSA. Although the protein concentration in the final dispersion decreased, the remaining proteins are expected to retain their amino acid sequence and not have extensive changes in their properties, such as their intrinsic pH and isoelectric points.

### Composition of amino acid residues

Recent reports suggest that amino acid residues, such as aspartic acid, cysteine, glutamic acid, tyrosine and tryptophan, can reduce Au(III) and Ag(I) ions [12,18,38,41–46]. In this study, amino acid content of each protein is different due to differences in their molecular weights and amino acid sequences. Thus, predicting that a particular amino acid is responsible for the reduction of Au^3+^ to Au^0^ is difficult. We calculated the relative percentages of each amino acid residue for all of the proteins based on their sequences and compared them with the reported compositions for HIS [46], LYS [47], OVA [48], BHG [49], BSA [50], TRY [51] and GOX [52] to determine which types of amino acids could be responsible for the formation of AuNPs. The percentage of hydrophobic residues (HR), aromatic residues (ArR), polar residues (PR), acidic residues (AcR), basic residues (BR), and charged residues (CR) contained in each protein was calculated and compared with the intensities of the SPR bands. The reactions conditions, such as temperature, microwave irradiation power and concentration of the reactants, were identical for all of the proteins. The diversity in the yield of AuNPs could mainly be due to the amino acid content and the structure of the protein during the reaction [53]. HIS, which led to a highly intense and narrow SPR band for the AuNP, and TRY, which did not form any AuNPs (no SPR band), possess similar molecular weights, thus comparing their amino acid content would be useful to understand the role of various amino acids residues that could be responsible for the formation of the AuNPs. Studies have revealed that proteins with a lower percentage of aromatic residues and a higher percentage of basic, charged, and polar residues favor the formation of AuNPs (Table S1, Supporting Information File 1). Disulfide bridges also play an important role in the yield of AuNPs during the reaction. From the UV–vis studies (Figure 1A and Figure 1B), the SPR bands of the AuNPs prepared by using LYS and BSA were broader with lower intensities compared to the other four proteins. No AuNPs were formed with TRY. This behavior could be due to the high number of disulfide bridges present in the proteins LYS, BSA and TRY (4, 17 and 6, respectively, Table 1).

#### Zeta potential studies

**Figure 3:**
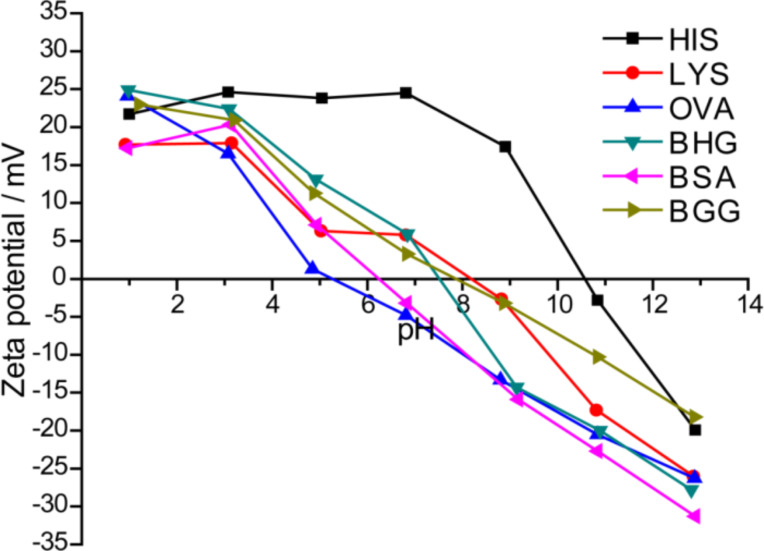
Variation in the zeta potential of AuNPs prepared by using different proteins as a function of the pH in aqueous medium.

The amino acid content of proteins leads to different isoelectric points (IEPs). IEP is the point at which the colloidal dispersion carries no charge and is least stable [54]. Therefore, to determine the IEPs of the protein-coated AuNPs, their aqueous dispersions were subjected to pH-dependent zeta potential titration studies. The AuNPs behavior in the human body can be understood by examining the correlation between pH and zeta potential. Figure 3 shows the results for the different protein-coated AuNPs. The zeta potentials at the intrinsic pH of the AuNPs were determined, and the values are in Table S2 (Suporting Information File 1). The IEPs of the AuNPs ranged from acidic to basic pH values. Although all the AuNPs possess an IEP value at which the colloidal dispersion is least stable we did not observe any agglomeration behavior for the AuNPs. The AuNPs prepared by using OVA had an IEP of 5.08, an acidic pH. A basic pH of 10.58 was measured for the HIS-AuNPs. The IEPs for remainder of the AuNPs were between the values. The IEP values suggest that HIS-AuNPs have a predominantly positive surface charge and that an excess of OH^−^ ions are required for the net surface charge to be zero. In contrast, the OVA-AuNPs reach a zero net surface charge at pH 5.08, thus carrying predominantly negative charges on the surface of the OVA-AuNPs at their intrinsic pH. The IEPs of the remaining four AuNPs were close to neutral and ranged from 6 to 8.5. To compare the results with blank proteins, three different proteins, LYS, BSA and BGG (different molecular weights), were subjected to titrations studies. From these studies, we observed that the zeta potential profiles for the blank protein and the AuNPs prepared by using LYS and BSA were similar. However, the profiles for BGG were slightly different (Figure S6, Supporting Information File 1). The difference in this zeta potential profile is because of the drastic change in the structural conformation of BGG during microwave irradiation, which was also observed in the UV–vis studies for the blank proteins (Figure S3, Supporting Information File 1).

#### Cell viability after exposure to AuNPs

**Figure 4:**
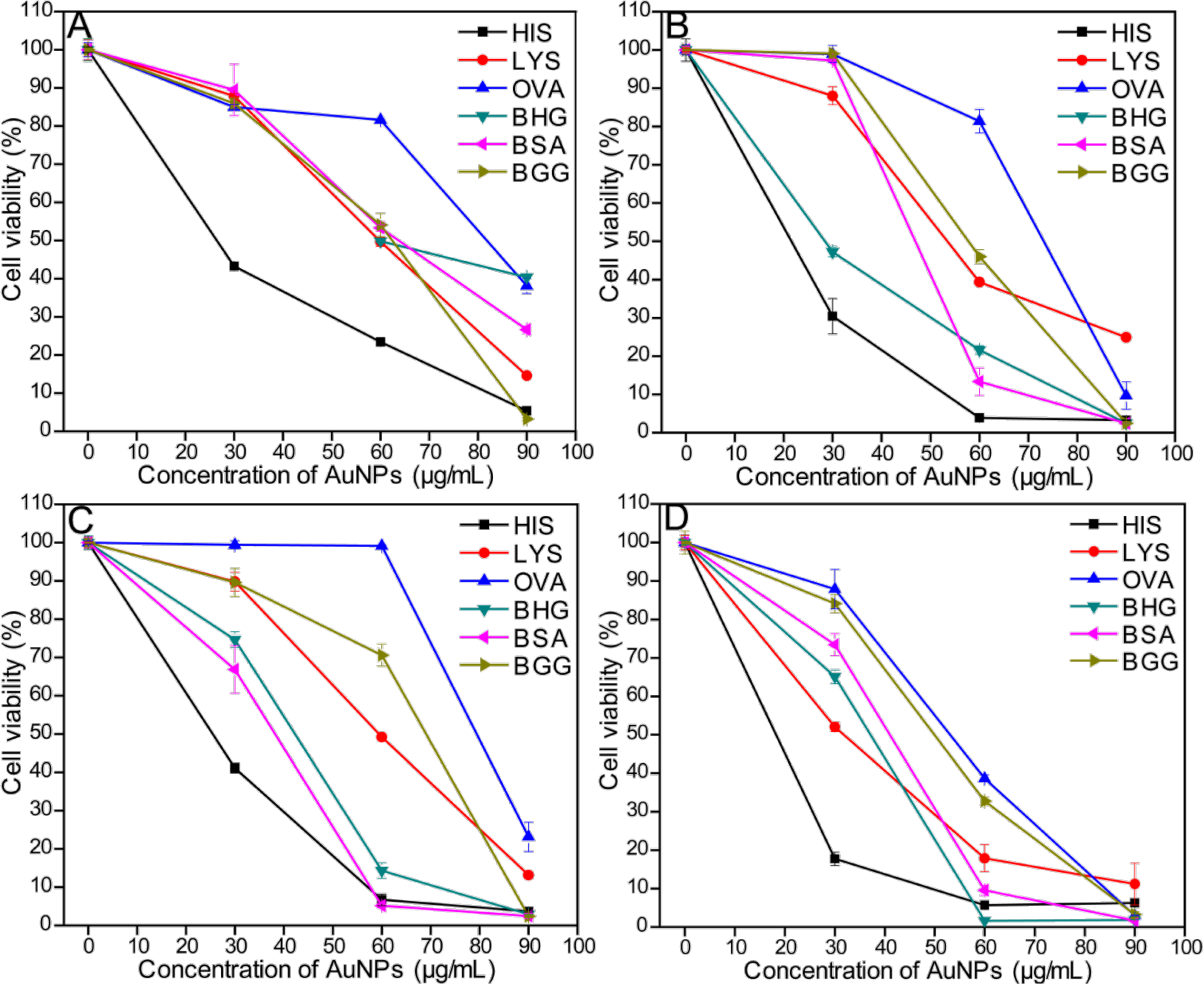
Cell viability studies by using MTT assay on (A) NIH-3T3, (B) HCT116, (C) HeLa and (D) SCC-7 cells treated with AuNPs prepared by using different proteins.

To investigate the cell viability after the exposure to AuNPs, MTT assays were conducted with mouse embryonic fibroblasts (NIH-3T3) by treating them with AuNPs samples prepared by using different proteins (Figure 4A). Exposure to AuNPs resulted in different cell viability values based on the protein used for capping. The OVA-coated AuNPs had the highest IC50 value, and HIS-AuNPs had the lowest. From the zeta potential titration studies (Figure 3), HIS-AuNPs had the highest IEP value of 10.58, which indicates that it carries a predominantly positive surface charge. The OVA-AuNPs had the lowest IEP value (5.08), indicating a predominant negative surface charge. The remaining four AuNPs had IEP values between 6 and 8.5. Correlating the IEP and the IC50 values of the different AuNPs revealed that the cell viabilities after exposure to the AuNPs decreased with increasing IEP values. Therefore, the HIS-AuNPs, which had an IEP value of 10.58, resulted in the lowest cell viability, leading to high toxicity. The OVA-AuNPs, which had an IEP value of 5.08, resulted in the highest cell viability, displaying lower toxicity at certain concentrations. The zeta potentials of AuNPs at their intrinsic pH also revealed a similar behavior (Table S2, Supporting Information File 1).

The mechanism involved in causing the differences in the cell viability of AuNPs coated by different proteins at fixed concentrations is speculated to be due to the degree of internalization of the AuNPs into the cells, which is supported here by the zeta potential studies. The internalization of AuNPs highly depends upon the surface charge of the AuNPs.

**Figure 5:**
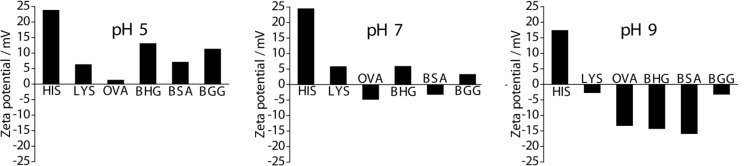
Zeta potential of AuNPs prepared using different proteins at pH 5, 7 and 9.

Thus, the surface charges on the AuNPs at a pH of 7 (physiological pH) were studied based on the pH-dependent zeta potential titration plot (Figure 5). The HIS-AuNPs had a large positive zeta potential that exceeded 20 mV, which corresponds to a positive surface charge. The OVA-AuNPs had a negative zeta potential that was close to zero and hence carried a negative surface charge. A recent report shows that gold nanospheres attached to a negatively charged cell surface penetrate the cell wall more easily, if they carry positive charges, because the cell membrane tries to restore its previous surface charge distribution by removing the attached charged particles through endocytosis or other methods [55]. Because of their higher positive surface charge, HIS-AuNPs are easily internalized by cells, after which they cause cell death. A recent report suggests that the interior of lysosomes possesses an acidic pH (pH 4–5), and it agglomerates the protein-coated AuNPs that enter into it by enzymatic digestion [56]. Since the as-prepared protein-coated AuNPs reported here are highly pH-sensitive we believe that the AuNPs are agglomerated inside the lysosomes thereby causing cytotoxicity. HIS is non-covalently bound to the AuNPs, and the protein can easily become detached from the AuNPs during this process, resulting in high toxicity to the cells. The plasma membrane contains fewer cationic sites on the suface than the anionic sites, but reports suggest the absorption of negatively charged nanoparticles [57–58]. A cluster of negatively charged nanoparticles are formed that binds randomly to the cationic sites, which then undergoes endocytosis [59]. In this study, exposure to OVA-AuNPs resulted in a high cell viability due to the poor ability of the nanoparticles to internalize into the cells because of their low level of cellular interaction. Those AuNPs that enter the cells do not affect it more than OVA, which contains a disulfide bridge that is broken during microwave irradiation. The breaking of the disulfide bridge allows two sulfur groups to bind to the AuNPs covalently, reducing the exposure of the surface of the AuNP to the cells. Exposure to OVA-AuNPs at higher concentrations resulted in a lower cell viability, which could be due to the agglomeration of the AuNPs in the interior of the lysosomes [56]. Among the other four protein-coated AuNPs, the zeta potential values were negative for BSA-AuNPs and positive for LYS-AuNPs, BHG-AuNPs and BGG-AuNPs. All of these AuNPs had zeta potentials close to zero; hence their IC50 values were also similar. The IC50 values of the different protein-coated AuNPs followed the order OVA > BSA > BHG > LYS > BGG > HIS for the NIH-3T3 fibroblasts.

**Figure 6:**
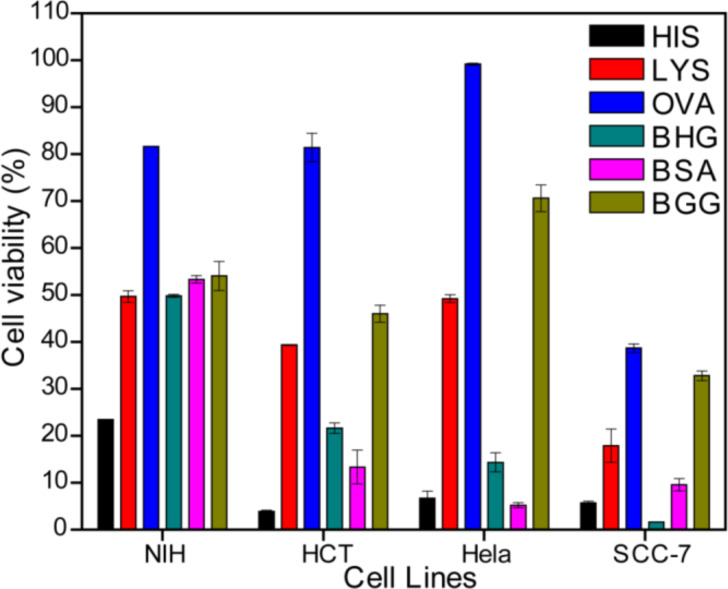
Plot representing cell viability of AuNPs prepared by using different proteins vs different cell lines at 60 µg/mL concentration of the AuNPs studied using MTT assay.

To study further the role of the prepared AuNPs as an effective anticancer agent, MTT assays were performed against three different cancer cells lines (human colorectal cancer cells (HCT116), human cervical cancer cells (HeLa) and squamous carcinoma cells (SCC-7)) by treating them with AuNPs. The results are shown in Figure 4B, 4C and 4D respectively. The surface charge on the AuNPs at an acidic pH was studied and we observed all the AuNPs possess a positive surface charge at pH 5. The HIS-AuNPs and OVA-AuNPs exhibited the same behavior for all three cancerous cell lines as with the NIH-3T3 cell line. The HIS-AuNPs were the most toxic, and OVA-AuNPs were the least toxic. Interestingly, the order was different for the other four protein-coated AuNPs. Certain protein-coated AuNPs were more toxic to cancerous cells than to the fibroblasts (see Table S3, Supporting Information File 1). For example, the IC50 for the BSA-AuNPs in NIH-3T3 was 68.20 µg/mL and 32.68 µg/mL for Hela, which shows that these nanoparticles cause 50% inhibition of the cancer cell at a lower concentration in comparison to the fibroblasts. This can be attributed due to the difference in the surface charge at different pH conditions. The surface charge on BSA-AuNPs at acidic pH (which is considered to be the pH in cancer cells) [60] was positive and negative at pH 7 (Figure 5). Hence higher cell internalization takes place in an acidic environment causing greater toxicity compared to neutral pH. The IC50 values of the different protein-coated AuNPs followed the order OVA > BGG > LYS > BSA > BHG > HIS, OVA > BGG > LYS > BHG > BSA > HIS and OVA > BGG > BSA > BHG > LYS > HIS for the HCT116, HeLa and SCC-7 cell lines, respectively (Table S3, Supporting Information File 1). The MTT assay on the blank proteins showed cell viabilities greater than 80% (Figure S7, Supporting Information File 1). The protein-coated AuNPs exhibited specificity towards certain cancer cell lines, which was observed from the differences in their IC50 values. The BHG-AuNPs has the second lowest IC50 value for HCT116, while the BSA-AuNPs and the LYS-AuNPs were the second lowest for the HeLa and SCC7 cell lines, respectively. The cell viability of AuNPs for different cell lines at a particular concentration (60 µg/mL) is represented in Figure 6 to highlight their behavioral differences and specificity. Hence, the appropriate concentration of the prepared AuNPs could be used for the treatment of cancer without damaging or causing less damage to the normal cells. We speculate that the difference in the order of the cell viabilities is due to the difference in the pH among the cancer cells as their origin is different. However, the specificity is unclear. The MTT assays revealed differential effect of AuNPs on the cell viabilities of the fibroblasts and the cancer cell lines. Additional studies, such as cellular uptake and in vivo studies must be conducted to unravel the mechanism involved in the internalization of AuNPs and to understand the anticancer properties of the prepared AuNPs.

## Conclusion

For the first time, six different protein-coated AuNPs were prepared by using a single, rapid and green synthetic protocol that utilized microwave irradiation in an aqueous medium. The addition of silver ions to the reaction mixture enhanced the yield of AuNPs, which was confirmed by a narrow and intense SPR band. The hydrodynamic sizes confirm the protein coating on the AuNPs. The protein coating affects their behavior in varying pH conditions. The AuNPs possesses different isoelectric points and zeta potentials, which account for the differences in the cell viability percentages and IC50 values observed in the MTT assays. By correlating the zeta potentials and the IC50 values of the AuNPs, we predict that the positively charged AuNPs are internalized by the cells to a greater extent than are the negatively charged AuNPs. The MTT assays revealed that certain AuNPs resulted in lower cell viability for cancerous cells in comparison to fibroblasts. Hence, increasing evidence from these studies is pointing that by controlling the concentrations of the AuNPs, anticancer agents can be formulated. These synthesized AuNPs show promise for use in biomedical applications for diagnostics and drug delivery due to their pH sensitivity, high stability and biocompatible surfaces.

## Experimental

### Materials and characterization

Potassium tetrachloroaurate (KAuCl_4_) was purchased from Aldrich. Silver nitrate (AgNO_3_) was obtained from Kojima Chemicals Co. Ltd., Korea. All of the proteins, calf histone (HIS), chicken egg white lysozyme (LYS), ovalbumin (OVA), bovine serum albumin (BSA), bovine hemoglobin (BHG), bovine gamma globulin (BGG), glucose oxidase (Aspergillus niger) (GOX), and trypsin (porcine pancreas, TRY), used for the experiments were obtained from Sigma (USA) as a lyophilized powder. All of the chemicals were used as received. Deionized (Milli-Q grade) water was used to prepare all of the solutions. A microwave (CEW, Discover Benchmate, CEW Co., Korea) system that allows microwave synthesis with safe pressure regulation by using a 10 mL pressurized vial and cap and automatically vents when the internal pressure of the vial reaches 300 psi was used for the preparation of the AuNPs. UV–vis spectra for the as-prepared AuNPs (0.5 mM) were recorded on a PerkinElmer UV–vis spectrophotometer (Lambda 750) as triplicates. Transmission electron microscopy (TEM) images were obtained with a JEOL JEM-2100 transmission electron microscope operating at 200 kV. The samples for the TEM analysis were prepared by placing a drop of the nanoparticle dispersion on carbon-coated copper grids and then drying in an oven at 60 °C for 12 h. The size of the AuNPs in the TEM images was measured by using the ImageJ software. The hydrodynamic size and zeta potential were recorded by light scattering using ELS 8000 (Photal, Otsuka Electronics Co. Ltd, Japan). Sodium dodecyl sulfate polyacrylamide gel electrophoresis (SDS-PAGE) was performed by using an electrophoretic apparatus (Amersham Biosciences). The BSA-AuNP sample was mixed with 2× sample buffer (Sigma) and heated at 80 °C for 15 min. Further protein was separated through a 4% stacking gel and 12% separation gel. To determine the amount of protein in the sample, the gels were stained with Coomassie blue (Sigma) staining solution for 2 h. The stained gels were washed with distilled water 2–3 times and further incubated with destaining solution (50% methanol and 10% acetic acid) overnight. On the following day, the gels were dried and scanned. The FASTA sequences for all of the proteins (P0C0S9, P00698, P01012, P01966&P02070, P02769, P00772 and P13006 for HST, LYS, OVA, BHG, BSA, TRY, and GOX, respectively) were downloaded from the UniProt protein database archive to calculate the compositions of the amino acid residues in the proteins.

#### Synthesis of protein-coated AuNPs

In a typical reaction, 5 mL of an aqueous solution containing 1 mg/mL of protein, KAuCl_4_ (0.025 mL, 0.1 M) and AgNO_3_ (0.005 mL, 0.1 M) was added to a 10 mL capped vial and placed in the microwave system at 250 W and 120 °C for 10 min. After this step, the reaction mixture was allowed to cool down to 25 °C before further characterizing the samples and performing the cytotoxicity studies. The reactions were also carried out in the absence of AgNO_3_, keeping all of the other conditions the same as above.

#### Cell culture and cell viability test (MTT assay)

In a similar manner to the methods described in [61], Mouse embryonic fibroblasts (NIH-3T3), human colorectal cancer cells (HCT116), and human cervical cancer cells (HeLa) were routinely maintained in Dulbecco’s Modified Eagles Medium (DMEM) supplemented with 10% fetal bovine serum (FBS, SIGMA) and 1% antibiotic/antimycotic (GIBCO). Squamous carcinoma cells (SCC-7) cell lines were grown in Roswell Park Memorial Institute Media (RPMI) 1640 (SIGMA) containing 10% FBS and 1% antibiotic-antimycotic. The NIH-3T3 and HeLa cells were originally obtained from the Korean Cell line Bank (Seoul, Korea). The SCC7 and HCT-116 cells were purchased from the American Type Culture Collection. All of the cells were cultured in a humidified atmosphere containing 5% CO_2_ and maintained at 37 °C. To assess the effect of AuNPs on the viability percentages of the cells, MTT {3-(4, 5-dimethylthiazol-2-yl)-2,5-diphenyltetrazolium bromide} colorimetric assays were performed. The cells were seeded in 96 well plates at a density of 5 × 10^4^ cells/well and incubated for 24 h in 200 µL of culture media at 37 °C. On the following day, the cells were further treated with different concentrations of blank proteins and AuNPs prepared in fresh media, substituting the prior culture media in which cells were seeded. After 24 h incubation, the media were removed, the cells were rinsed with PBS, and the cells were again incubated with 40 µL of MTT solution for 4 h. The supernatant was removed carefully, and dimethyl sulfoxide (Junsei Chemical Co. Ltd) was then added to each well to dissolve the purple formazan produced by the MTT. The absorbance was measured by using a microplate reader (FL600, Microplate Fluorescence Reader, Bio-Tek Company) at a wavelength of 570 nm. The concentration of the sample causing 50% inhibition of proliferation of the cells (IC50) was determined by plotting the percentage of cell viability versus the sample concentrations [62]. All experiments were carried out in triplicates.

## Supporting Information

Supporting Information features tables containing amino acid compositions in proteins, IEP and IC50 values of AuNPs, UV–vis spectral studies and photographs of colloidal dispersions on the preparation of AuNPs at acidic, neutral and basic pH conditions, structural conformation studies by using UV–vis, FTIR and SDS-PAGE, zeta potential of AuNPs in comparison with the blank proteins, and cell viability studies by using MTT assays on cells treated with different proteins.

File 1 Additional experimental data.
