# Supplementary material for: Protein-coated pH-responsive gold nanoparticles: Microwave-assisted synthesis and surface charge-dependent anticancer activity
Source: Beilstein J Nanotechnol. 2014 Sep 4;5:1452–62. doi: 10.3762/bjnano.5.158 (PMC4168932; doi:10.3762/bjnano.5.158)
Supplement: File 1 — Additional experimental data. [file Beilstein_J_Nanotechnol-05-1452-s001.pdf]

Supporting Information

for

Protein-coated pH-responsive gold nanoparticles: Microwave-assisted synthesis and surface charge-dependent anticancer activity

Dickson Joseph[1], Nisha Tyagi[1], Christian Geckeler[2] and Kurt E.Geckeler*[1,2,3,§]

Address: [1]Department of Nanobio Materials and Electronics (WCU), Gwangju Institute of Science & Technology (GIST), Gwangju 500-712, South Korea; [2]Department of Materials Science and Engineering, Gwangju Institute of Science & Technology (GIST), Gwangju 500-712, South Korea and [3] Institute of Medical System Engineering, Gwangju Institute of Science & Technology (GIST), Gwangju 500-712, South Korea.

E-mail: Kurt E.Geckeler* - keg@gist.ac.kr

* Corresponding author

[§]Fax: (+82-62-715-2338) Tel: (+82-62-715-2316)

Additional experimental data

Table S1: Composition of amino acid residues in each protein.[a]

Amino Acid Residue	HIS	LYS	OVA	BHG	BSA	TRY
	Proteins (%)					
Hydrophobic (V, I, L, M, F, W,C)	24.61	33.68	38.58	34.94	30.85	33.45
Aromatic (F, W, Y, H)	8.5	15.25	14.18	19.56	14.93	16.81
Basic (R, K, H)	30.24	18.32	13.65	20.5	20.26	11.87
Acidic (E, D)	8.13	7.42	13.67	11.71	18.36	5.09
Charged (R, K, E, D)	34.44	24.78	25.09	25.14	35.12	13.79
Polar (R, K, D, E, N, Q)	43.95	38.61	34.08	30.46	41.37	28.68

[a]The amino acid residues, R: Arginine, N: Asparagine, D: Aspartic Acid, C: Cysteine, Q: Glutamine, E: Glutamic Acid, H: Histidine, I: Isoleucine, L: Leucine, K: Lysine, M: Methionine, F: Phenylalanine, W: Tryptophan, Y: Tyrosine, V: Valine.

Table S2: pH, I.E.P, zeta potential and size of the AuNPs prepared using different proteins.

Protein	pH	I.E.P	Zeta potential(mV)	Size distribution (nm)	
				DLS	TEM
HIS	3.23	10.58	49.09	20.7 ± 5.9	9.16
LYS	3.03	8.40	40.06	35.1 ± 10.3	13.20
OVA	3.24	5.08	31.15	27.4 ± 7.6	10.85
BHG	3.35	7.56	38.81	28.2 ± 7.6	13.46
BSA	2.97	6.16	33.92	31.0 ± 8.7	6.91
BGG	2.82	7.88	23.84	21.3 ± 6.1	7.59

Table S3: IC50 values of the AuNPs prepared using different proteins for four cell line variants.

Proteins	IC50 (μg/mL)			
	NIH-3T3	HCT-116	Hela	SCC-7
HIS	28.87	20.6	23.9	17.86
LYS	63.32	59.94	63.5	30
OVA	82.27	74.11	80.48	57.25
BHG	66.94	30.2	39.7	30.86
BSA	68.2	49.5	32.68	37.19
BGG	62.8	63.4	68.6	52.9

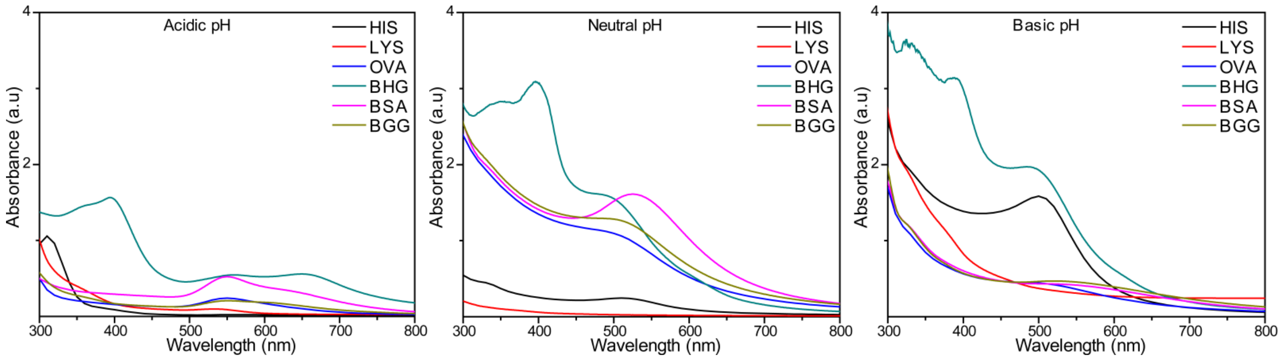

Figure S1: UV-Vis spectral studies on the preparation AuNPs at acidic, neutral and basic pH conditions.

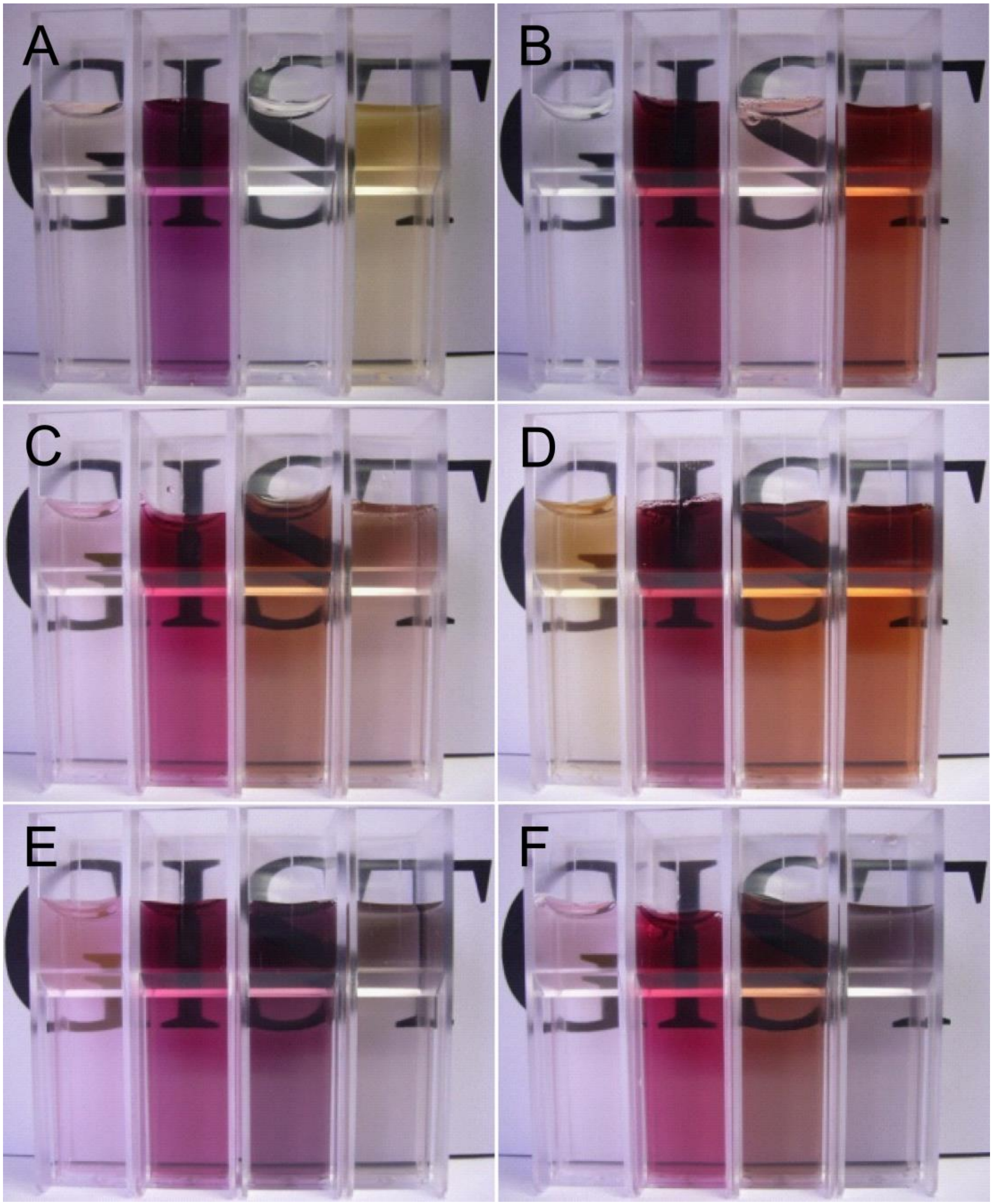

Figure S2: Photographs of the dispersions after the microwave irradiation under different pH conditions; acidic, intrinsic, neutral and basic (left to right) for reaction carried out using different proteins: (A) HIS, (B) LYS, (C) OVA, (D) BHG, (E) BSA and (F) BGG.

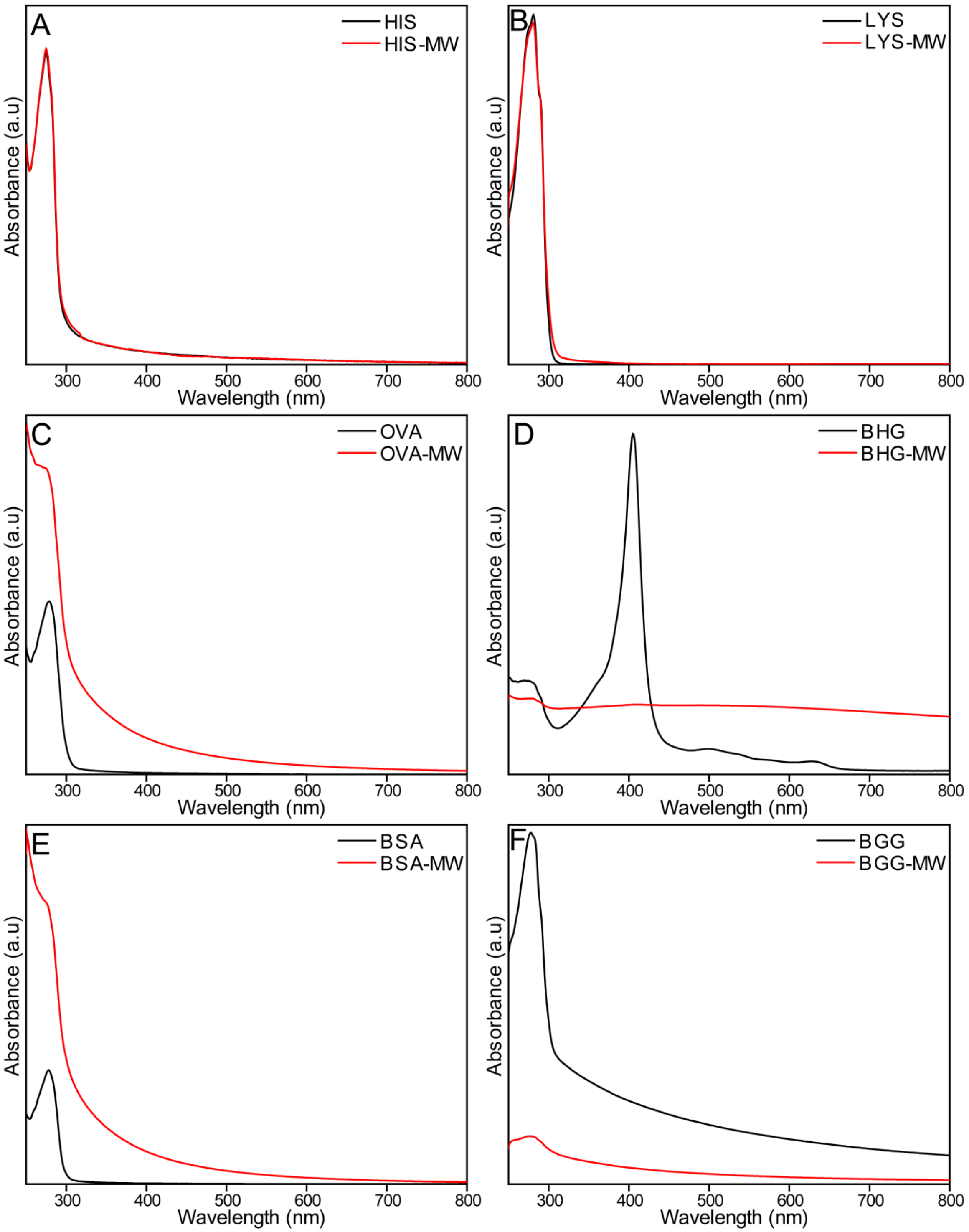

Figure S3: UV-Vis spectra of different blank proteins: (A) HIS, (B) LYS, (C) OVA, (D) BHG, (E) BSA and (F) BGG that where microwave irradiated and non-irradiated.

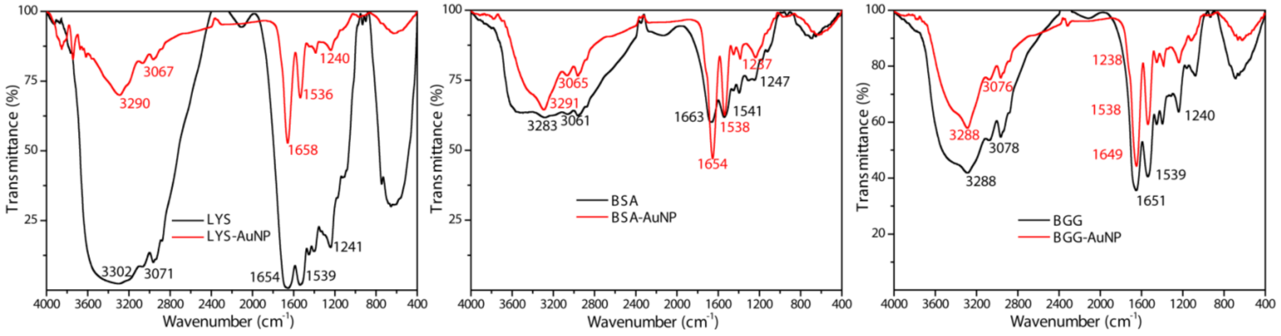

Figure S4: FT-IR spectra of blank proteins (black) and AuNPs prepared using corresponding proteins (red).

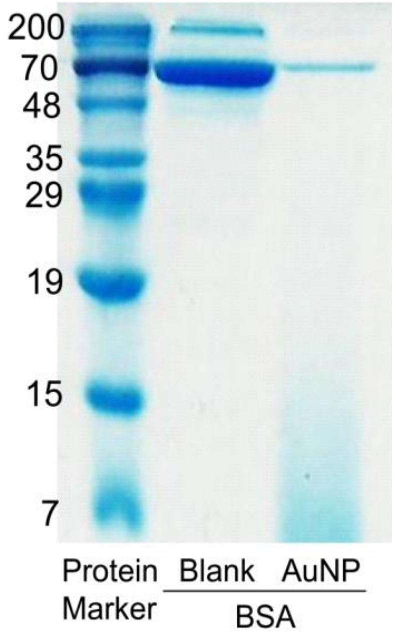

Figure S5: Electrophoresis profile of BSA in a gel after the synthesis of AuNPs as followed by SDS-PAGE; molecular weight marker (lane 1), blank BSA (lane 2), AuNPs prepared using BSA (lane 3).

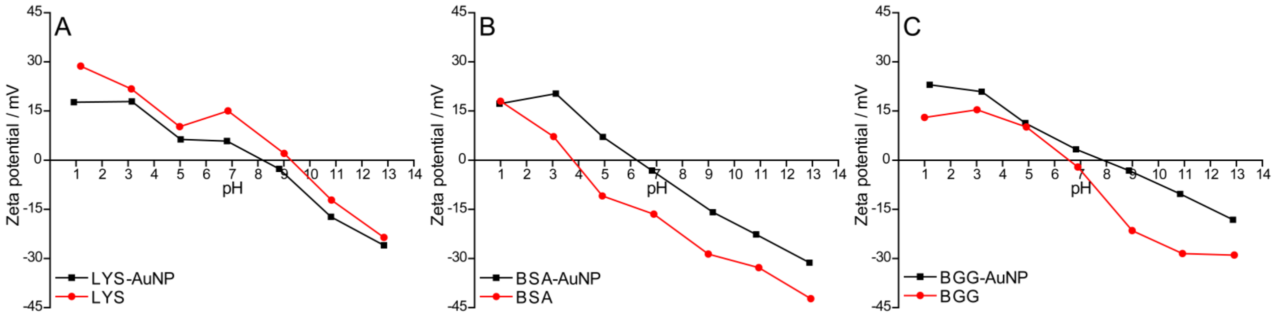

Figure S6: Variation in the zeta potential of AuNPs and blank proteins (A) LYS, (B) BSA and (C) BGG as a function of the pH in aqueous medium.

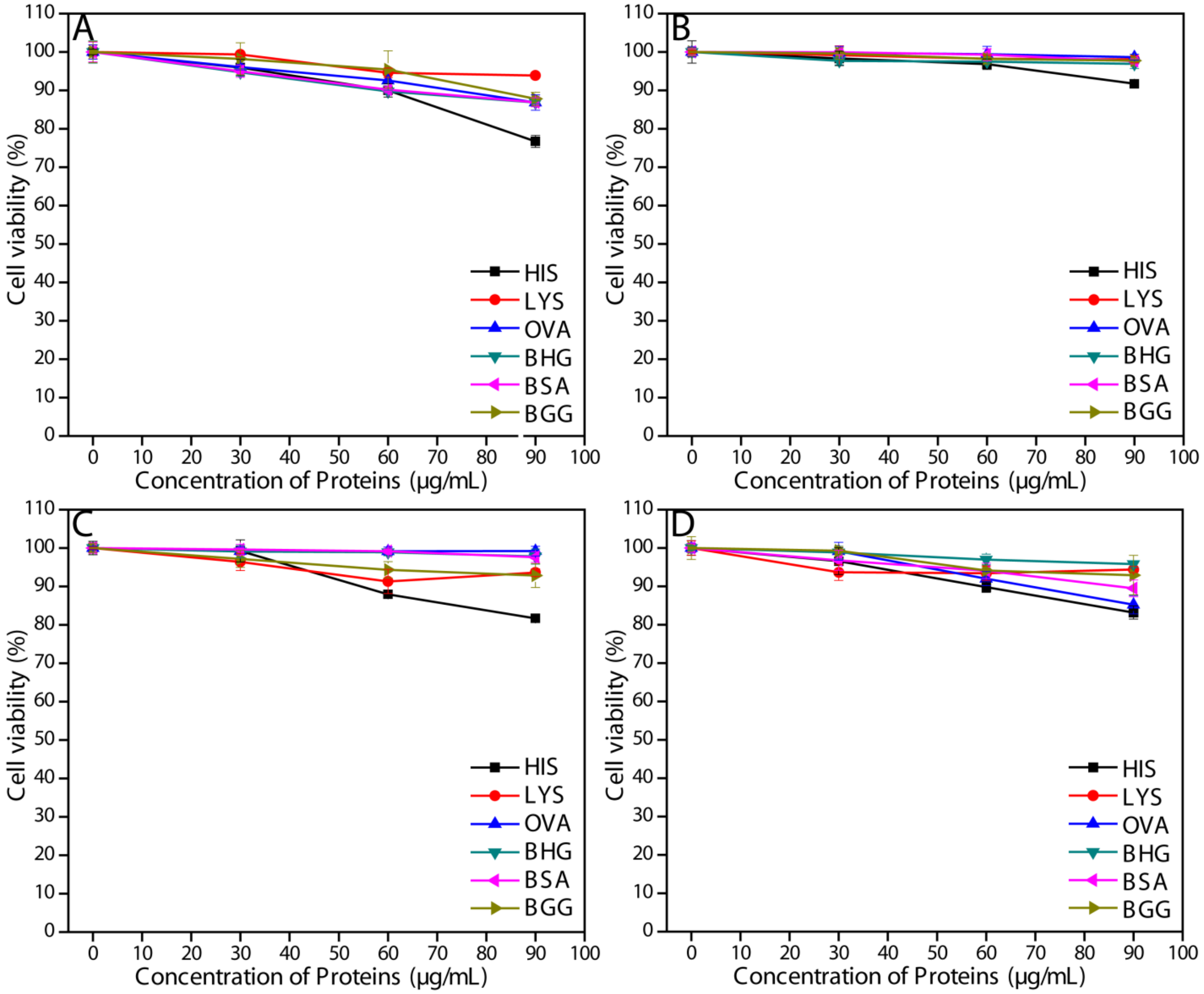

Figure S7: Cell viability studies using MTT assay on (A) NIH-3T3, (B) HCT116, (C) HeLa and (D) SCC7 cells treated with different blank proteins.
